# Neural and Behavioral Correlates of Clinical Improvement to Ketamine in Adolescents With Treatment Resistant Depression

**DOI:** 10.3389/fpsyt.2020.00820

**Published:** 2020-08-18

**Authors:** Michelle Thai, Zeynep Başgöze, Bonnie Klimes-Dougan, Bryon A. Mueller, Mark Fiecas, Kelvin O. Lim, C. Sophia Albott, Kathryn R. Cullen

**Affiliations:** ^1^ Psychology Department, College of Liberal Arts, University of Minnesota, Twin Cities, MN, United States; ^2^ Department of Psychiatry and Behavioral Sciences, School of Medicine, University of Minnesota, Twin Cities, MN, United States; ^3^ Biostatistics Department, School of Public Health, University of Minnesota, Twin Cities, MN, United States

**Keywords:** depression, adolescence, ketamine, fMRI, affective conflict, treatment resistance, emotional stroop

## Abstract

Treatment-resistant depression (TRD) is a serious problem in adolescents. Development and optimization of novel interventions for these youth will require a deeper knowledge of the neurobiology of depression. A well-established phenomenon of depression is an attention bias toward negativity and away from positivity that is evidenced behaviorally and neurally, but it is unclear how symptom reduction is related to changes to this bias. Neurobiological research using a treatment probe has promise to help discover the neural changes that accompany symptom improvement. Ketamine has utility for such research because of its known rapid and strong antidepressant effects in the context of TRD. Our previous study of six open-label ketamine infusions in 11 adolescents with TRD showed variable response, ranging from full remission, partial response, non-response, or clinical worsening. In this study, we examined the performance of these participants on Word Face Stroop (WFS) fMRI task where they indicated the valence of affective words superimposed onto either congruent or incongruent emotional faces before and after the ketamine infusions. Participants also completed a clinical assessment (including measurement of depression symptomology and anhedonia/pleasure) before and after the ketamine infusions. Following ketamine treatment, better WFS performance correlated with self-reported decreased depressive symptoms and increased pleasure. Analyses of corticolimbic, corticostriatal and default mode (DMN) networks showed that across networks, decreased activation during all conditions (congruent negative, congruent positive, incongruent negative, and incongruent positive) correlated with decreases in depressive symptoms and with increases in pleasure. These findings suggest that in adolescents with TRD, clinical improvement may require an attenuation of the negativity bias and re-tuning of these three critical neural networks to attenuate DMN and limbic regions activation and allow more efficient recruitment of the reward network. Lower activation across conditions may facilitate shifting across different salient emotional stimuli rather than getting trapped in downward negative spirals.

## Introduction

Depression, the second leading cause of disability globally, commonly arises during the adolescent period (1). While evidence-based treatments are available, such as cognitive behavioral therapy and selective serotonin inhibitor antidepressants, up to 40% of adolescents with depression fail to respond to these interventions (2), emphasizing the need to discover new efficacious treatments. Development and optimization of novel interventions will require a deeper knowledge of the neurobiology of depression and the key neural changes that accompany treatment response.

Examination of neural and behavioral indices in the context of a clinical trial provides the opportunity to identify the key brain changes that accompany clinical improvement. Ketamine has utility for such research because of its known rapid and strong antidepressant effects with adults who are suffering from depression (3–6). In our recent pilot study testing open-label ketamine in adolescents with depression, there was significant variability in response at 2 weeks, ranging from full remission, partial response, non-response, or clinical worsening (7). Although we had expected more consistently positive outcomes, this variability provides an advantage for identifying neurobehavioral correlates of symptom improvement.

Examination of neural correlates of clinical improvement with ketamine treatment should build on existing knowledge about the neural systems implicated in depression. To date, neuroscience research investigating the neural underpinnings of depression has demonstrated disturbances in key neural networks including the corticolimbic, corticostriatal, and default mode (DMN) networks. In corticolimbic circuitry, limbic regions involved in the experience of negative emotion, such as the amygdala and subcallosal cingulate, are hyperactive (8–10). In contrast, striatal regions, which mainly respond to positive features of the environment, are underactive in depression (11–14). The DMN represents a group of brain regions that are more active at rest and less active during a demanding cognitive task (15). In depression, DMN hyperactivation is thought to reflect perseverative, negative, self-focused, ruminative thoughts. Hence, failure to disengage DMN activation during a task might interfere with task performance (16).

### Cognitive and Neural Underpinnings of Depression

A well-established phenomenon of depression that relates to all three of these neural systems is the negativity bias, in which negative information captures attention at the expense of positive information (17). This phenomenon can be successfully captured in Emotional Stroop tasks, in which individuals with depression are found to be quicker at detecting negative targets but have difficulty disengaging from negative distractors (18, 19). Individuals with depression also show a lack of a positivity bias (hence a difficulty in engaging with positive stimuli) (17, 20–22). The presence of a negativity bias and the lack of a positivity bias may account for patients with depression failing to consistently show an emotional interference effect (slowing to emotionally-loaded conflicting stimuli) as healthy controls do (23–26). For patients with depression, negative distractors are effective in drawing attention away from positive targets but positive distractors are ineffective in disengaging attention from negative targets. The cumulative result of these two biases is a failure to show a significant emotional interference effect. There is evidence that error rates from Emotional Stroop tasks also demonstrate a negativity bias: patients with depression are most accurate in incongruent conditions with negative targets and positive distractors (23, 24, 27).

Prior functional magnetic resonance imaging (fMRI) research assessing the negativity bias using Emotional Stroop tasks has implicated the corticolimbic, default mode, and corticostriatal networks in individuals with depression. Corticolimbic dysfunction underlies the rapid detection and more in-depth processing of negative stimuli demonstrated by patients with depression, as well as their difficulty inhibiting this automatic processing of negative stimuli. For example, in the Emotional Stroop task, patients with depression show greater activation in limbic regions irrespective of congruency, but lower lateral prefrontal cortex activation in response to emotional conflict than controls (28). DMN dysfunction during the Emotional Stroop is reflected by overactivity of the right precuneus compared to controls, which correlates with longer delays in responding to negative words (29). It may be that negative words elicit ruminative thoughts in patients with depression, causing abnormal DMN activity during the task and representing another illustration of the negativity bias in patients with depression. Although the corticostriatal network has not been highlighted as much in Emotional Stroop fMRI research, some evidence suggests its importance in how positive mood states interact with behavioral and neural responses during the task (30). While these neural and behavioral characteristics of the negativity bias in depression are now well-established, their dynamics with respect to course of illness and clinical improvement are still unknown.

### Current Study

In this pilot study, we used ketamine as a probe to discover the behavioral and neural changes associated with depressive symptom improvement in adolescents with treatment-resistant depression (TRD). Specifically, we examined behavioral performance and neural activation in three key networks (corticolimbic, corticostriatal, and DMN) within the context of an Emotional Stroop task (the valence-specific Word-Face Stroop [WFS] Task) during MRI scans that took place before and after a series of ketamine infusions. In the WFS, participants evaluated the valence of emotionally laden words overlaid on congruent or incongruent emotional faces (24). Specifically, we examined how clinical improvement was linked with behavioral and neural changes measured during the WFS. Behaviorally, we hypothesized that clinical improvement would correlate with a reduction in the negativity bias such that participants would attend more to positive targets and distractors, and less to negative targets and distractors. Neurally, we hypothesized that clinical improvement would be associated with a reduction in corticolimbic and DMN activation to negative stimuli and increased corticostriatal activation to positive stimuli. In addition to providing preliminary evidence of behavioral and neural activation changes accompanying symptom changes in TRD, the findings may have implications for clarifying possible mechanisms of ketamine treatment.

## Materials and Methods

### Participants

Thirteen adolescents with treatment-resistant depression (TRD) completed a clinical trial investigating intravenous ketamine as a treatment for adolescent depression [see (7) for clinical results and details about treatment procedures and tolerance]. This study was approved by the University of Minnesota Institutional Review Board. All participants (or their parents for those under 18 years of age) gave written informed consent or assent (for participants 12 to 17 years of age) in accordance with the Declaration of Helsinki. Of these 13 participants, two were excluded due to technical errors in collecting their WFS data, resulting in a final sample of 11 participants (age: *M* = 17.02, *SD* = 1.18, range: aged 14 to 18; 3 female) with TRD. Participants were recruited through clinical referrals and community postings. Inclusion criteria included a current diagnosis of Major Depressive Disorder (MDD), a Children’s Depression Rating Scale-Revised [CDRS-R (31)] raw score >40, stable psychotherapy for 2 months (Years in therapy: *M* = 3.26; *SD* = 2.66), stable psychotropic medication dose for 2 months, and evidence of treatment resistance. Treatment resistance was defined as failure to respond to a minimum of two antidepressant medications as assessed by the Antidepressant Treatment History Form (32). All participants have participated in psychotherapy (dialectical behavioral therapy: *n* = 4; cognitive behavioral therapy: *n* = 4; supportive: *n* = 2; unknown: *n* = 1). Exclusion criteria included current substance use disorder, primary psychotic disorder, bipolar disorder, autism spectrum disorder, intellectual disability, a neurological disorder, or significant medical illness.

### Procedures

Participants completed a baseline visit that included informed consent and/or assent (for children under 18), a diagnostic interview, and clinical measures of depression symptomatology. Participants also completed a magnetic resonance imaging (MRI) scan at baseline. Following baseline visits, participants who met inclusion criteria received six ketamine infusions across 2 weeks (see (7) for more details about ketamine infusions and assessment schedule). Post-treatment assessments, including a post-treatment MRI scan, were completed 1 day following the last ketamine infusion. In contrast to our other work (7), this study is not intended to assess the impact of ketamine treatment, but rather to identify neural correlates of clinical improvement of ketamine treatment in this sample of youth with TRD.

### Measures and Tasks

#### Psychiatric Diagnoses

The Kiddie Schedule for Affective Disorders and Schizophrenia-Present and Lifetime Version [KSADS-PL; (33)], a semi-structured diagnostic interview, was used to diagnose Axis I disorders based on the *Diagnostic and Statistical Manual of Mental Disorders* [4^th^ ed., text rev.; (34)]. Trained clinical psychologists, child psychiatrists, or advanced clinical psychology doctoral students supervised by a senior clinician conducted independent KSADS-PL interviews with adolescents and parents. Consensus was established between adolescent and parent reports and was used to determine diagnoses. All participants had a diagnosis of MDD.

#### Depression Symptoms

Examiners also assessed for depression symptoms using the Children’s Depression Rating Scale-revised [CDRS-R; (31)] and the Montgomery–Åsberg Depression Rating Scale [MADRS; (35)]. CDRS-R scores were based on consensus between parent and child reports. CDRS-R percent change was calculated using the formula: (baseline raw CDRS-R - posttreatment raw CDRS-R)/(baseline raw CDRS-R - 17) (36). Larger scores represent more improvement over the course of treatment. We examined associations with post-treatment minus pre-treatment MADRS scores to supplement findings from the CDRS-R; these results are presented in the **Supplementary Materials**.

#### Anhedonia and Pleasure

Participants completed a self-report measure of pleasure, the Temporal Experience of Pleasure Scale [TEPS; (37)]. Higher TEPS scores reflect greater degrees of experienced pleasure. Change in TEPS scores were calculated by subtracting baseline TEPS scores from post-ketamine TEPS scores. Larger values indicate an increase in pleasure. We focus on the TEPS total score. TEPS anticipatory (TEPS-A) and consummatory pleasure (TEPS-C) subscale total scores showed similar patterns of results (see **Supplementary Materials**).

#### Word-Face Stroop Task

The Word Face Stroop (24) is a valence specific Emotional Stroop task (originally in Turkish) that has shown to successfully work as an emotional conflict resolution task (23, 24, 27). Unlike previous word-face Emotional Stroop tasks (38–41), the WFS isolates the emotional conflict to the valence dimension by eliminating the arousal dimension and using emotionally laden words (e.g., “toy,” “ulcer”) instead of emotion names (e.g., “anger,” “happiness”) (see (24) for additional reasoning behind these selections). In the English version of the task, 32 positive (valence mean = 7.27 ± 0.43; arousal mean = 4.84 ± 0.39) and 32 negative target words (valence mean = 2.59 ± 0.52; arousal mean = 4.74 ± 0.33) with neutral arousal levels were selected from ANEW (42). The distracter affective human faces (4 happy and 4 sad; 2 male and 2 female in each emotion category) were selected from The Productive Aging Lab Face Database (43).

**Figure 1:**
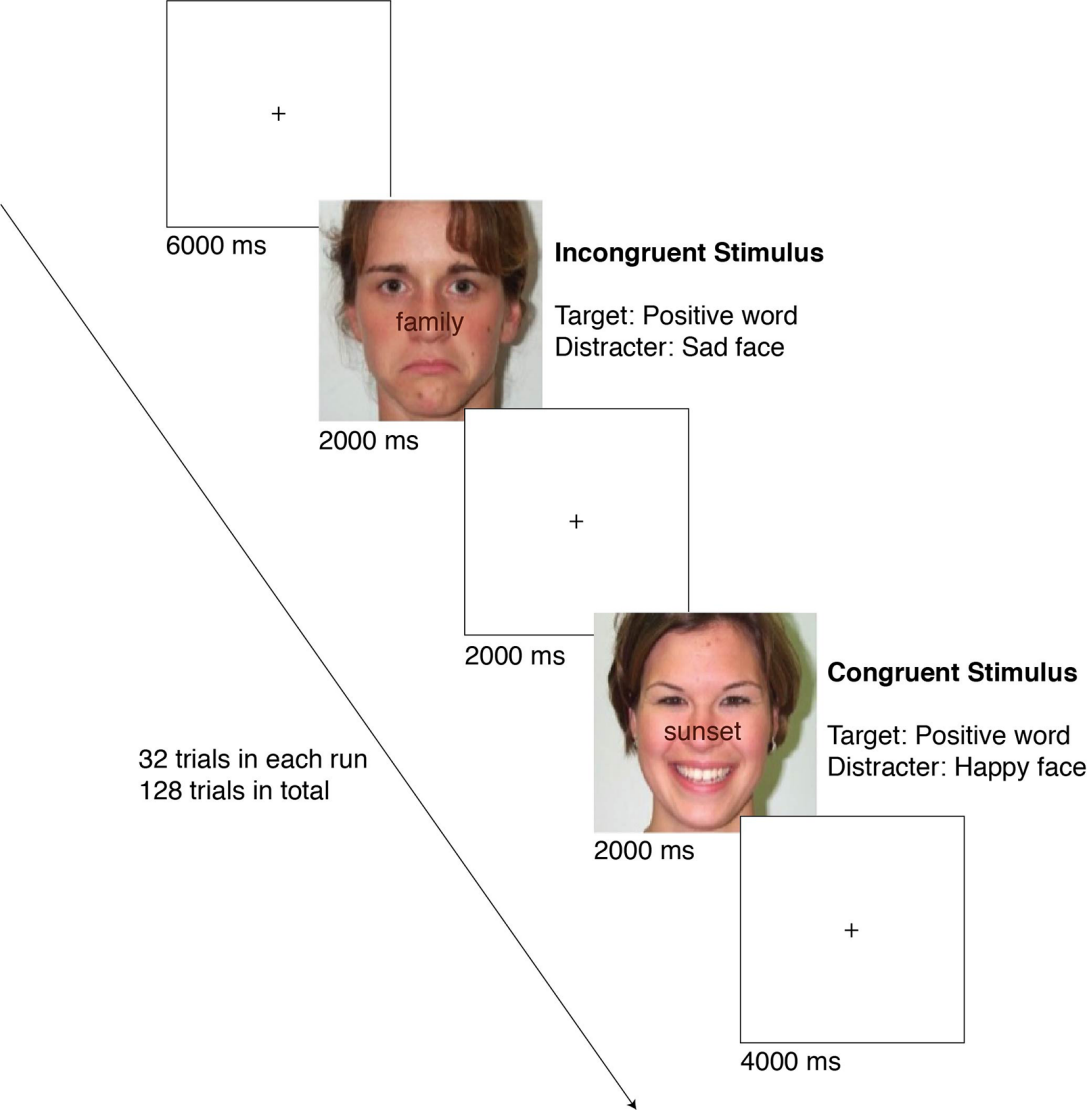
The flow of an experiment session. The examples show an incongruent positive (top) and a congruent positive (bottom) case.

Participants were asked to evaluate the valence of the word (as positive or negative) superimposed on an affective face (happy or sad). Participants had 2,000 ms to respond before the next trial appeared. A fixation cross was presented for 2, 4, or 6 s in-between trials. In order to prevent boredom effects, participants completed four separate 32-trial runs in the scanner (128 trials in total). There were two main independent variables, each having two levels of conditions: congruency (congruent *vs.* incongruent) and valence (positive *vs.* negative). In congruent trials, the valence of the word and face matched and in incongruent trials, the valence of the word and face differed. A condition’s valence is dependent upon the valence of the word and not the face. Therefore, in positive trials, the word was positive, and in negative trials, the word was negative, irrespective of the valence of the face, which works as the distractor. According to picture superiority effect, the emotion of the picture is expected to be prominent while the emotion of the word is faint (44, 45) such that the face works as a distractor. Hence, the subjects are supposed to inhibit the prominent emotion as induced by the face and respond to the emotion carried by the word. Thus, there were a total of four different embedded conditions: Congruent Positive (positive word over happy face), Congruent Negative (negative word over sad face), Incongruent Positive (positive word over sad face), and Incongruent Negative (negative word over happy face). The flow of an experiment session is summarized in **Figure 1**. Dependent variables include reaction time and accuracy (correct response rate [CRR]).

### Neuroimaging

#### Data Acquisition

Neuroimaging data were acquired using a 3T Siemens Prisma scanner at the Center for Magnetic Resonance Research (CMRR) at the University of Minnesota (UMN). We utilized a multiband echo planar imaging (MB-EPI) sequence to improve the spatial and temporal resolution of the acquired fMRI data over conventional methods (46). Individual fMRI data (Multiband Factor [MB] = 3, Repetition Time [TR] = 2,000 ms [this specific TR was necessary because the task was programmed to count TR triggers in order to advance], Echo Time [TE] = 30 ms, 2 mm isotropic voxel size, 100 volumes [~3.5 min per run]), along with a B0 field map and high resolution T1 weighted MPRAGE anatomical scan (Magnetization-Prepared Rapid Gradient-Echo, TR = 2,530 ms, TE = 3.65 ms, Inversion Time [TI] = 1,100 ms, 7 degree flip angle, 1 mm isotropic voxel size, 4 min) were collected before and after the ketamine intervention for all 13 study participants.

#### fMRI Preprocessing and Processing

fMRI data were preprocessed using FEAT (FMRI Expert Analysis Tool) Version 6.00, part of FSL (FMRIB’s Software Library, www.fmrib.ox.ac.uk/fsl). A distortion map was computed using a reverse phase encode gradient echo scan and topup. Data were motion corrected using MCFLIRT (47). Brain extraction was conducted using BET2 (48). B0 field-map unwarping and distortion correction were applied using FUGUE. Registration to standard MNI space was carried out using FLIRT and a 12 degrees of freedom affine registration (47, 49). Spatial smoothing was applied using a Gaussian kernel of FWHM 3 mm.

Brain activity during the WFS task was analyzed using FSL. A first-level analysis was conducted to regress the WFS task model onto fMRI data for each individual run, using the four contrasts described above (Congruent Positive, Congruent Negative, Incongruent Positive, and Incongruent Negative). A second-level fixed effects analysis was conducted to combine results across runs (within each of the pre and post treatment sessions) for each participant. To isolate fMRI signal independent of behavioral performance, we included mean-centered average reaction time in each run as a covariate. Pre-treatment contrast maps were subtracted from post-treatment contrast maps to obtain a neural map representing the change in neural activity during the task after ketamine treatment. Z-scores were extracted from pre-defined regions of interest (ROIs) within the corticolimbic, corticostriatal, and default mode networks from these post-pre z-score maps. ROIs for the corticolimbic network include the left and right hippocampus, left and right amygdala, subcallosal cortex, and the anterior cingulate cortex (ACC). ROIs for the corticostriatal network include the left and right accumbens. ROIs for the DMN include the precuneus and the posterior cingulate cortex (PCC). ROIs were created using FSL with the Harvard-Oxford Cortical Structural Atlas and the Harvard-Oxford Subcortical Structural Atlas.

### Statistical Analyses

To measure change over time on the Word-Face Stroop Task behavioral performance, we conducted two 2×2×2 repeated measures ANOVA with congruency (congruent/incongruent), valence of the word (positive/negative), and time (pre/post) for reaction times and accuracy. To examine associations between clinical, behavioral, and neural changes, we conducted a series of Pearson’s correlations. For the clinical measures we considered CDRS-R percent change and post-treatment minus pre-treatment TEPS scores. To examine the relation between the changes in the clinical measures and in the task performance, we also calculated post-ketamine minus pre-ketamine performance (for accuracy and reaction time) for the main conditions (congruency and valence) and for the embedded conditions (congruent positive, congruent negative, incongruent positive, incongruent negative). For brain activation, we focused on the embedded conditions.

Given the limited power due to small sample size, a Bonferroni correction was not feasible for correlation analyses. Instead of focusing on p-values, we considered results to be important if they had strong effect sizes. The following guidelines were used to determine the strength of effect size: 0.1 to 0.3 represent a small to medium effect, 0.3 to 0.5 represent a medium to large effect, and >0.5 represent a large to very large effect size (50). We also conducted exploratory prediction analyses to examine the association between clinical change and baseline brain activation (see **Supplementary Materials**).

## Results

### Clinical Change Correlations

CDRS-R percent change was positively correlated with change in TEPS total score (*r* = .73, *p* = .01). CDRS-R percent change was strongly correlated with MADRS post minus pre change (*r* = −.94, *p* <.001), so we focused on CDRS-R percent change in the following analyses. Change in TEPS-A and TEPS-C were strongly positively correlated (*r* = .85, *p* = .001), so we focused on TEPS total score in the following analyses.

### Behavioral Performance

#### Task Performance

As would be expected with the WFS task, a repeated measures ANOVA on reaction times revealed a significant **congruency effect**, *F*(1,10) = 14.54, *η2* = 0.59, *p* < 0.01, meaning that participants were significantly slower towards incongruent cases, regardless of the condition. However, contrary to predictions, there was no significant main effect of valence or time (pre/post) or any significant interactions. The repeated measures ANOVA on accuracy revealed no significant results.

### Correlations Between Change in WFS Task Performance and Clinical Change

#### Main Conditions

Correlations between change in the CDRS-R and TEPS and change in the main conditions of congruence and valence for the WFS are shown on **Table 1**. A decrease in RT to congruent conditions on the WFS showed medium to very large correlations with improved depression on the CDRS-R and pleasure on the TEPS. A decrease in RT to the positive condition on the WFS showed a medium to large correlation with improved pleasure. An increase in accuracy on the incongruent condition and positive condition on the WFS showed medium to very large correlations with improved depression and pleasure. An increase in accuracy across the congruent condition and negative condition on the WFS also showed medium to very large correlations with improved pleasure.

**Table 1:** Change in Behavior and Clinical Change.

	CDRS-R % Change	TEPS Total Change
Congruent RT	−0.37	−0.42
Incongruent RT	0.11	−0.04
Positive RT	−0.09	−0.38
Negative RT	−0.26	0.01
Congruent Accuracy	0.19	0.58
Incongruent Accuracy	0.42	0.62*
Positive Accuracy	0.48	0.68*
Negative Accuracy	0.05	0.37
Congruent Positive RT	−0.27	−0.61*
Congruent Negative RT	−0.32	−0.10
Incongruent Positive RT	−0.36	−0.58
Incongruent Negative RT	0.20	0.23
Congruent Positive Accuracy	0.33	0.68*
Congruent Negative Accuracy	−0.06	0.25
Incongruent Positive Accuracy	0.58	0.58
Incongruent Negative Accuracy	0.14	0.46

Numbers represent Pearson’s Correlations. *indicates p <.05.

#### Embedded Conditions

**Figure 2:**
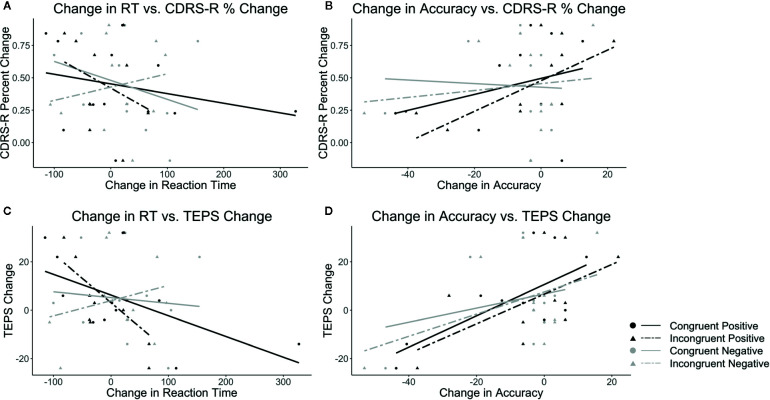
Plots show the correlations between CDRS-R percent change (Higher scores correspond to a greater reduction in depression) and **(A)** change in RT and **(B)** change in accuracy as well as correlations between TEPS total change (Higher scores correspond to a greater increase in pleasure) and **(C)** change in RT and **(D)** change in accuracy. Black lines indicate positive target word valence and gray lines represent negative target word valence. Solid lines represent congruent conditions and dashed lines represent incongruent conditions.

In terms of the embedded conditions (congruence and valence considered together), a decrease in RT to the congruent positive condition and incongruent positive condition on the WFS was associated with improved depression on the CDRS-R and pleasure on the TEPS. A decrease in RT to the congruent negative condition on the WFS was associated with improved depression. An increase in accuracy on the congruent positive and the incongruent positive conditions on the WFS was associated with improved depression and pleasure. Also, an increase in accuracy to the congruent negative and incongruent negative conditions on the WFS was associated with improved pleasure. See **Table 1** and **Figure 2**.

### Neural Activation

#### Correlations Between Change in ROI Activation With Clinical Change

After ketamine treatment, a decrease in activation in regions within corticolimbic and corticostriatal circuits across conditions showed medium to very large correlations with improved depression and pleasure. This pattern was predicted for regions within the corticolimbic but not the corticostriatal networks. The right accumbens and amygdala and subcallosal cortex in particular are largely consistent across conditions. Although this relationship was found across all conditions on the WFS, the regions in the corticolimbic and corticostriatal networks showed the strongest correlations in the congruent negative and incongruent negative conditions. The right hippocampus, however, showed the opposite pattern in the congruent positive condition with increased activation in the congruent positive condition being related to improved depression and pleasure.

**Figure 3:**
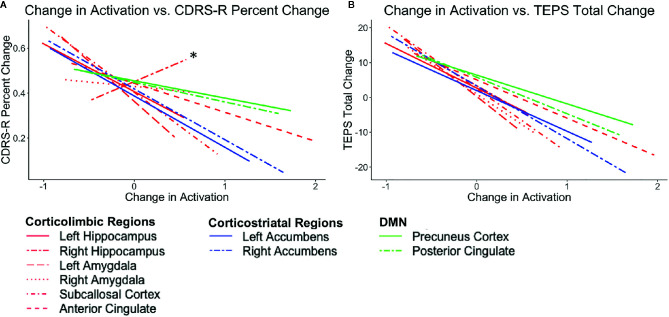
Plots show the correlations between the average post-pre change in activation (z scores) across conditions (congruent negative, congruent positive, incongruent negative, and incongruent positive) and **(A)** CDRS-R percent change (Higher scores correspond to a greater reduction in depression) and **(B)** TEPS total change (Higher scores correspond to a greater increase in pleasure). Lines in red correspond to corticolimbic regions; lines in blue correspond to corticostriatal regions, and lines in green correspond to DMN regions. * indicates the right hippocampus.

As predicted, decreased activation in regions associated with the DMN in the incongruent negative and incongruent positive conditions on the WFS showed small to large correlations with improved depression. Decreased activations in regions associated with the DMN across all four conditions on the WFS showed small to very large correlations with improved pleasure. The strongest relationships were found in the incongruent negative condition on the WFS. See **Figure 3** and **Table 2**. See **Supplementary Figure 3a** and **3b** for plots with data points.

**Table 2:** Change in Activation and Clinical Change.

Condition	Congruent Negative		Congruent Positive		Incongruent Negative		Incongruent Positive	
Neural Network/Brain Region	CDRS-R % Change	TEPS Total Change	CDRS-R % Change	TEPS Total Change	CDRS-R % Change	TEPS Total Change	CDRS-R % Change	TEPS Total Change
**Corticostriatal Network**								
Left Accumbens	−0.50	−0.54	−0.40	-0.32	-0.53	-0.48	-0.42	-0.54
Right Accumbens	−0.34	−.60*	−0.41	-0.42	-.61*	-.63*	-0.26	-0.43
**Default Mode Network**								
Posterior Cingulate	−0.04	−0.30	−0.05	-0.25	-0.32	-0.51	-0.20	-0.36
Precuneus	−0.04	−0.28	0.01	-0.15	-0.26	-0.45	-0.26	-0.32
**Corticolimbic**								
Left Amygdala	−0.35	−0.59	−0.10	−0.06	−0.60	−0.53	−0.27	−0.30
Left Hippocampus	−0.22	−0.44	−0.16	−0.03	−0.47	−0.45	−0.29	−0.41
Right Amygdala	−0.06	−0.53	−0.06	−0.31	−0.09	−0.41	0.05	−0.12
Right Hippocampus	0.07	−0.40	0.42	0.16	0.03	−0.39	0.12	−0.06
Subcallosal Cingulate	−0.39	−.62*	−0.34	−0.31	−0.53	−0.49	−0.39	−0.56
Anterior Cingulate	−0.15	−0.40	−0.30	−0.53	−0.46	−0.52	−0.24	−0.45

Numbers represent Pearson’s Correlations. *indicates p < .05.

## Discussion

This study investigated the behavioral and neural correlates of clinical improvement using the Word Face Stroop task (WFS) in adolescents with TRD within the context of a ketamine treatment trial. Overall, there was evidence that the WFS task parameters operated as expected in that conflicting information was processed more slowly than consistent information (main effect of congruency). However, this sample of adolescents with TRD did not demonstrate a negative affective bias as measured by reaction times or accuracy on the WFS. Importantly, the results of this study provide preliminary evidence that improvement in behavioral performance on the WFS was associated with a decrease in depression and an increase in pleasure. Consistent with expectations, a decrease in activation in regions within the corticolimbic and DMN networks when engaged in the WFS correlated with reductions in depression symptoms and increased self-reported pleasure. Contrary to expectations, this decrease was not specific to conditions with negative stimuli but rather found across all four conditions, and the same pattern was unexpectedly found for activation within the corticostriatal network.

Behaviorally, we predicted that in the context of ketamine treatment, clinical improvement would be related to a reduction in the negativity bias and an increase in positivity bias on the WFS. Although we did not find evidence of an overall reduction in the negativity bias, we found evidence of a link between improved performance for positive stimuli on the WFS and decreases in clinician-rated depression symptoms and increases in self-reported pleasure. Specifically, for the incongruent positive condition (where the target word is positive and the distractor face is sad), an improvement in performance (faster RT and greater accuracy) was associated with improved depression and pleasure. Past research shows that patients with depression perform worse on the incongruent positive condition, whereas they perform most accurately on the incongruent negative condition (where the target word is negative and the distractor face is happy), because they have trouble disengaging from negative stimuli and engaging with positive stimuli, either as a target or as a distractor (23, 24, 27). Therefore, the current findings supporting a link between increased accuracy (especially to the incongruent positive condition) and clinical improvement may suggest that attentional reserves have shifted toward positive stimuli and away from negative stimuli. By attending more to positive stimuli, greater opportunities for pleasure and reward may be sought, aiding in breaking out of anhedonia and experiencing more pleasure. Our lack of findings with respect to a behavioral change in the negativity bias may not be surprising given that we did not find evidence of a negativity bias at baseline. While meta-analyses have shown a negativity bias for depressed participants, the overall effect may be relatively weak as there is heterogeneity in findings with some studies failing to show negativity bias (17). Instead, our results may provide support for the cognitive efficiency theory. This theory suggests that individuals with depression may perform comparably with healthy individuals, in this case, not slowing in response to negative distractors, but in order to not show this bias, they may have to recruit additional resources (51).

Neurally, we hypothesized that a larger post-treatment reduction in activation in regions within the corticolimbic and DMN to negative stimuli would be related to clinical improvement. This hypothesis was partially supported, but lacked specificity. That is, the same pattern of greater post-treatment reduction in activation in corticolimbic and DMN regions being associated with clinical improvement was found across all four conditions and was not specific to conditions with negative valenced stimuli. Given the event-related design of the task, participants may not have fully disengaged from previous trials that may have contained negative stimuli even if the current trial did not (e.g., in the congruent positive trial). Likewise, controlling for average reaction time in the model in order to isolate the neural signal from individual differences in response time may have reduced differences across conditions. This pattern of association suggests that for clinical response, it may be necessary to reduce engagement of the corticolimbic and DMN regions. Reduced corticolimbic and DMN activation may decrease engagement with negative features of the environment, which may diminish perseverative negative perceptions about the self, the world, and the future. This pattern of decreased activation in key brain regions being associated with symptom improvement is a pattern of biological changes that is common to a range of antidepressant treatments (52, 53), including ketamine (54).

In terms of the corticostriatal network, we predicted a larger post-ketamine increase in activation in the nucleus accumbens to positive stimuli would be related to clinical improvement. Our results, however, showed the opposite pattern with a larger decrease in activation across all conditions on the WFS being related to greater clinical improvement. Ketamine has also shown to blunt striatal activation during reward tasks (55) in HC. Ketamine treatment may reestablish proper functioning of the reward-related network such that reward-related regions are activating more efficiently. On the color-word Stroop task, patients with depression exhibited greater ACC activation compared to healthy controls (56), suggesting that individuals with depression must overrecruit neural resources to perform comparably with healthy controls. Although these findings are not in line with the literature on reward network functioning and depression (57), they suggest that efficient reward network activation is more critical to the experience of pleasure rather than overall reward network activation. Recent work has suggested that antidepressant treatment is associated with more efficient temporal and parietal activation to an n-back task (58). In this case, more efficient reward network recruitment may preserve more resources for more in-depth processing of positive features of the environment by prefrontal regions.

This overall pattern of greater clinical improvement being associated with a reduction in activation across all three networks may suggest that TRD is associated with overall inefficient recruitment of brain regions. Post-intervention dampening of neural activation may allow participants to better respond to shifting task demands as trial parameters shift across the task as opposed to perseveration (DMN hyperactivation) on negativity (corticolimbic hyperactivation) and missed reward opportunities (corticostriatal hyperactivation). Less activation may allow resources to be more quickly shifted to other brain regions as needed for a task at hand. This greater task flexibility may translate outside of the laboratory to represent greater flexibility in response to changing situations in contrast to a depressive pattern of getting trapped by negativity.

Reduced perseveration and greater cognitive and neural flexibility hold important implications for suicidality, which is a concerning symptom of depression (59). Joiner’s interpersonal theory of suicide attributes suicide risk largely to three factors: perceived burdensomeness, thwarted belongingness, and acquired capability for suicide (60). Hyperactivity across the three aforementioned networks may have relevance to this theory of suicidality; for example, through maintaining persistent negative self-reflective thoughts about burdensomeness and lack of social connectedness. Notably, hyperactivity in brain regions across these three networks are implicated in social evaluation (61). Likewise, suicidality and self-injury are associated with limbic hyperactivity (62). Our finding that a decrease in activation across these three networks correlated with clinical improvement may indicate a restoration of normative function of these networks such that individuals are able to more readily disengage from negative self- and interpersonal cognitions. Testing the relevance of these network changes for reducing suicide risk requires further study.

The results of this study raise the possibility that this pattern of behavioral and neural findings is attributable to ketamine treatment for adolescents with TRD. Prior fMRI research in patients with depression has shown that ketamine treatment was associated with a reduction in overactivation in regions of the DMN (mPFC, precuneus, PCC), as well as in frontal (ACC) and limbic regions (insula) during an emotional face processing task (63) as well as reduction of subgenual cingulate overactivation during an incentive-processing task (54). Improvement in depressive symptoms in the context of a ketamine treatment trial was associated with a decrease in amygdala and left parahippocampal activation to angry faces and an increase in amygdala/left parahippocampal gyrus activation to happy faces (64). Ketamine treatment has also been associated with increased corticostriatal activity to positive faces [caudate; (65)]. Ketamine also increases prefrontal global connectivity in patients with MDD (66), which may suggest that ketamine acts to restore prefrontal regulatory control over limbic, DMN, and reward regions to mitigate the negativity bias.

On an important note, findings of the current study need to be replicated in a randomized, placebo-control trial before determining any causal conclusions about the effects of ketamine. An important issue to consider in future research is to what extent these results pertain to a specific treatment per se as opposed to clinical improvement that may take place in any number of treatments or even in the context of spontaneous remission. Here, we are limited to drawing preliminary conclusions about the associations of behavioral and neural patterns that are associated with changes in depression and pleasure. Additionally, without a healthy control group, it is unclear whether ketamine treatment restored normal healthy behavioral or neural patterns of responding. Although our sample included adolescents with treatment-resistant depression, it is unclear whether these findings are unique to TRD or whether these findings would extend to other depressive disorders. There are several other imitations to this pilot study. Most notably, the small sample size was a major limitation. Although we showed moderate to large correlations between clinical, behavioral, and neural indices in the context of ketamine treatment, only 11 adolescents had usable WFS task data. Accordingly, the study findings reported here preclude strong conclusions about the behavioral and neural evidence of clinical change. Time-lapse research is needed to understand the longevity of these observed changes for behavioral and neural indices and whether these changes are temporary or permanent, especially if future efforts were to find that these results are attributable to ketamine treatment, given that the effects of ketamine are expected to be relatively short-lived (approximately 1 to 2 weeks; (3)). Further, given the unexpected association between clinical improvement and a reduction in reward-related brain activity, ketamine treatment effects should also be examined specifically in the context of a reward task to better understand the role of the corticostriatal network in depression and in recovery from depression. Other issues that will need to be addressed include limited evidence of WFS validity, design idiosyncrasies [e.g., dosage strategy changed midway as noted in (7)], and constraints on external validity (e.g., primarily males were participants).

In conclusion, the findings of this pilot study identify preliminary evidence for the behavioral and neural correlates of clinical improvement in adolescents with treatment-resistant depression. Improved performance on the WFS, particularly in the context of positive conditions, was associated with clinical improvement. Neurally, consistent across all four WFS conditions, following ketamine treatment, a decrease in activation in corticolimbic, corticostriatal, and DMN networks was associated with a reduction in depression symptoms and an increase in pleasure. Clinical improvement may require reducing the negativity bias by increasing attention to positivity and retuning these three critical neural networks to attenuate hyperactivation in corticolimbic and DMN regions and allow more efficient recruitment of the reward network. Lower activation across these emotion eliciting conditions may facilitate shifting across different salient emotional stimuli rather than getting stuck in downward negative spirals. This work may have implications for not only how treatment works but examining new methods of treating youth who suffer from TRD. Interventions that reduce the negativity bias and limbic hyperactivity, like attention bias modification, may aid in prolonging the antidepressant effects of ketamine (67). Neurofeedback may also hold promise for extending intervention effects by training populations with depression to decrease neural hyperactivity (68). Finally (although as noted in **Supplemental Tables 2** and **4** we did not identify behavioral markers at baseline that predicted treatment response but we did identify neural predictors), determining personalized approaches to treating TRD is also a promising new frontier in mental health treatment research.

## Data Availability Statement

The datasets generated for this study are available on request to the corresponding author.

## Ethics Statement

The studies involving human participants were reviewed and approved by the University of Minnesota Institutional Review Board. Written informed consent to participate in this study was provided by the participants’ legal guardian/next of kin.

## Author Contributions

KC, BK-D, KL, CA, and BM were involved in planning and conducting the study. MT and ZB carried out the data analyses. MF was involved in developing the data analysis strategy. BM carried out fMRI data preprocessing. MT, ZB, KC, and BK-D were involved in drafting of the manuscript. All authors contributed to the article and approved the submitted version.

## Funding

This research was supported by the National Institutes of Health’s National Center for Advancing Translational Sciences (UL1TR002494, 1UL1RR033183, UL1TR000114), Biotechnology Research Center (P41 EB015894), the NINDS Institutional Center Core Grants to Support Neuroscience Research (P30 NS076408), the High Performance Connectome Upgrade for Human 3T MR Scanner (1S10OD017974-01), and the University Foundation, Amplatz Scholarship.

## Conflict of Interest

The authors declare that the research was conducted in the absence of any commercial or financial relationships that could be construed as a potential conflict of interest.
