# Supplementary material for: Neural and Behavioral Correlates of Clinical Improvement to Ketamine in Adolescents With Treatment Resistant Depression
Source: Front Psychiatry. 2020 Aug 18;11:820. doi: 10.3389/fpsyt.2020.00820 (PMC7461781; doi:10.3389/fpsyt.2020.00820)
Supplement: Supplementary file 1 [file DataSheet_1.docx]

Supplementary Material

Neural and behavioral correlates of clinical improvement to ketamine in adolescents with treatment resistant depression

**Michelle Thai**^1^**, Zeynep Başgöze**^2^**, Bonnie Klimes-Dougan**^1^**, Bryon A. Mueller**^2^**, Mark Fiecas**^3^**, Kelvin O. Lim**^2^**, C. Sophia Albott**^2^**, & Kathryn R. Cullen**^2^

^1^1Psychology Department, College of Liberal Arts, University of Minnesota, Twin Cities, MN, USA.

^2^Department of Psychiatry and Behavioral Sciences, School of Medicine, University of Minnesota, Twin Cities, MN, USA.

^3^Biostatistics Department, School of Public Health, University of Minnesota, Twin Cities, MN, USA.

*** Correspondence:**Michelle Thai, S474 Elliot Hall, Department of Psychology, 75 East River Road, Minneapolis, MN 55455

email - thaix049@umn.edu

**Keywords: depression, adolescence, ketamine, fMRI, affective conflict, treatment resistance, emotional stroop.**

# Supplementary Figures and Tables

**Behavioral Performance**

**Correlations between change in WFS task performance and clinical change.**

MADRS post-pre change shows a similar pattern (but opposite sign) of correlations as the CDRS-R % change. TEPS-A and TEPS-C subscales showed a similar pattern of correlations as the TEPS Total score. See Supplementary Table 1 and Supplementary Figure 1.

| Supplementary Table 1. Change in Behavior and Clinical Change | | |  |  |  |
| --- | --- | --- | --- | --- | --- |
|  | CDRS % Change | MADRS  Change | TEPS-A  Change | TEPS-C  Change | TEPS Total Change |
| Congruet RT | -0.37 | 0.45 | -0.35 | -0.51 | -0.42 |
| Incongruent RT | 0.11 | -0.02 | -0.06 | 0.00 | -0.04 |
| Positive RT | -0.09 | 0.24 | -0.32 | -0.45 | -0.38 |
| Negative RT | -0.26 | 0.24 | 0.00 | 0.01 | 0.01 |
| Congruent Accuracy | 0.19 | -0.27 | 0.49 | .68* | 0.58 |
| Incongruent Accuracy | 0.42 | -0.43 | 0.52 | .74** | .62* |
| Positive Accuracy | 0.48 | -0.46 | .62* | .72* | .6 |
| Negative Accuracy | 0.05 | -0.15 | 0.27 | 0.54 | 0.37 |
| Congruent Positive RT | -0.27 | 0.38 | -.62* | -0.51 | -.61* |
| Congruent Negative RT | -0.32 | 0.43 | -0.02 | -0.25 | -0.10 |
| Incongruent Positive RT | -0.36 | 0.43 | -0.55 | -0.59 | -0.58 |
| Incongruent Negative RT | 0.20 | -0.19 | 0.17 | 0.33 | 0.23 |
| Congruent Positive Accuracy | 0.33 | -0.38 | .65* | .66* | .68* |
| Congruent Negative Accuracy | -0.06 | -0.03 | 0.13 | 0.46 | 0.25 |
| Incongruent Positive Accuracy | 0.58 | -0.49 | 0.50 | .69* | 0.58 |
| Incongruent Negative Accuracy | 0.14 | -0.24 | 0.38 | 0.57 | 0.46 |
| Numbers represent Pearson’s Correlations. * indicates p < .05 | | | |  |  |

Supplementary Figure 1

Supplementary Figure 1. Plots show the correlations between MADRS change, TEPS-A Change, and TEPS-C Change and change in RT and change in accuracy. Black lines indicate positive target word valence and gray lines represent negative target word valence. Solid lines represent congruent conditions and dashed lines represent incongruent conditions.


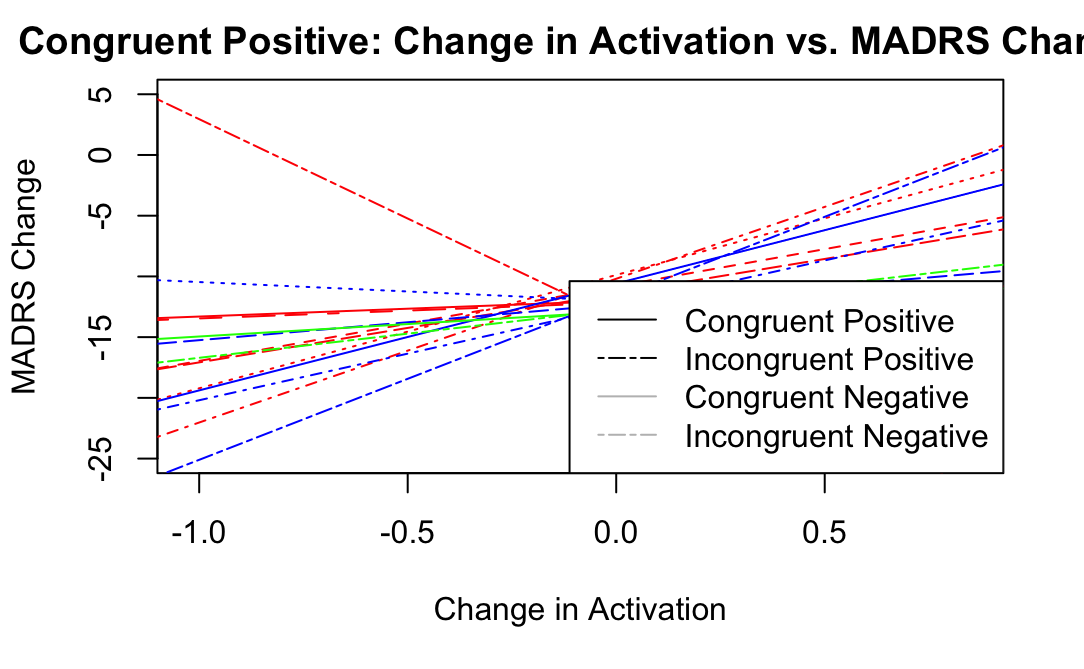


**Correlations between baseline WFS task performance and clinical change.**

There were no significant relationships between baseline WFS performance and clinical change. See Supplementary Table 2 and Supplementary Figure 2.

| Supplementary Table 2. Baseline Behavior and Clinical Change | | | |  |  |
| --- | --- | --- | --- | --- | --- |
|  | CDRS % Change | MADRS  Change | TEPS-A  Change | TEPS-C  Change | TEPS Total Change |
| Congruent RT | 0.13 | 0.01 | 0.08 | 0.04 | 0.07 |
| Incongruent RT | 0.15 | 0.03 | 0.08 | 0.01 | 0.06 |
| Positive RT | -0.01 | 0.13 | 0.08 | 0.01 | 0.06 |
| Negative RT | 0.25 | -0.06 | 0.06 | 0.03 | 0.05 |
| Congruent Accuracy | 0.05 | -0.07 | -0.14 | 0.04 | -0.09 |
| Incongruent Accuracy | -0.15 | 0.08 | -0.17 | -0.09 | -0.15 |
| Positive Accuracy | -0.06 | -0.02 | -0.25 | -0.07 | -0.20 |
| Negative Accuracy | -0.07 | 0.05 | -0.05 | -0.01 | -0.04 |
| Congruent Positive RT | 0.02 | 0.10 | 0.11 | 0.03 | 0.09 |
| Congruent Negative RT | 0.17 | 0.00 | -0.05 | -0.03 | -0.04 |
| Incongruent Positive RT | 0.10 | 0.06 | 0.02 | -0.02 | 0.01 |
| Incongruent Negative RT | 0.06 | 0.12 | -0.12 | -0.13 | -0.13 |
| Congruent Positive Accuracy | 0.14 | -0.17 | -0.26 | -0.01 | -0.18 |
| Congruent Negative Accuracy | -0.05 | 0.03 | 0.01 | 0.06 | 0.02 |
| Incongruent Positive Accuracy | -0.19 | 0.08 | -0.23 | -0.11 | -0.19 |
| Incongruent Negative Accuracy | -0.08 | 0.05 | -0.09 | -0.05 | -0.08 |
| Numbers represent Pearson’s Correlations. * indicates p < .05 | | | | |  |

Supplementary Figure 2

Supplementary Figure 2. Plots show the correlations between CDRS-R percent change, MADRS change, TEPS total score change, TEPS-A, and TEPS-C Change and baseline RT and baseline accuracy. Black lines indicate positive target word valence and gray lines represent negative target word valence. Solid lines represent congruent conditions and dashed lines represent incongruent conditions.


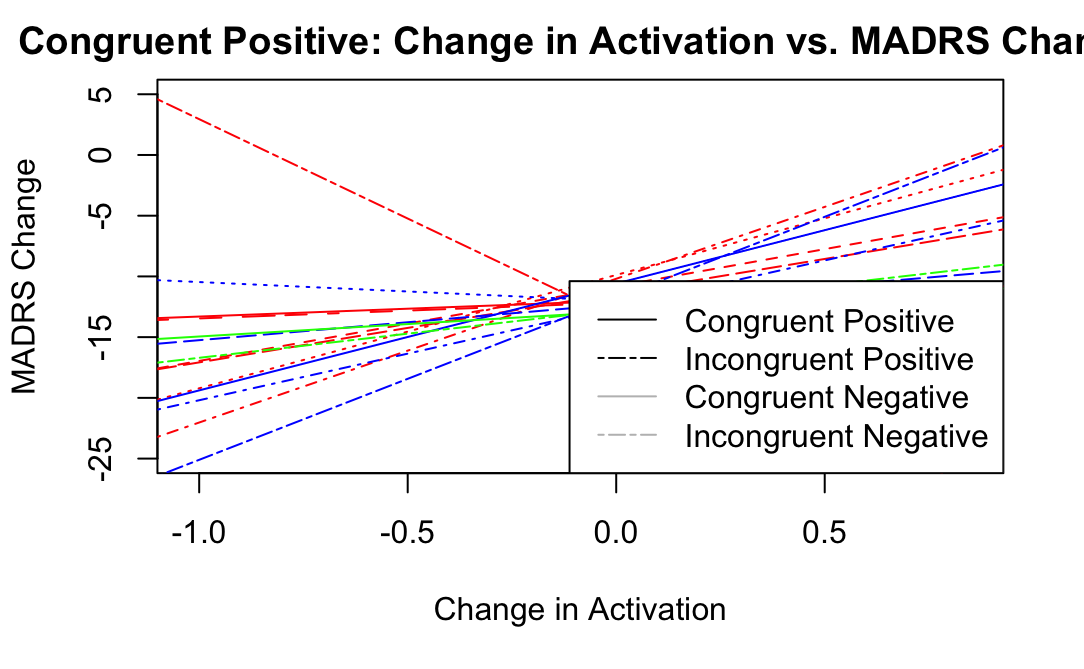


**Neural Activation**

Supplementary Figure 3a

Supplementary Figure 3a. Plots show the correlations between the average post-pre change in activation (z scores) across conditions (congruent negative, congruent positive, incongruent negative, and incongruent positive) and CDRS-R percent change (Higher scores correspond to a greater reduction in depression). Lines in red correspond to corticolimbic regions; lines in blue correspond to corticostriatal regions, and lines in green correspond to DMN regions. * indicates the right hippocampus.

Supplementary Figure 3b

Supplementary Figure 3b. Plots show the correlations between the average post-pre change in activation (z scores) across conditions (congruent negative, congruent positive, incongruent negative, and incongruent positive) and TEPS total change (Higher scores correspond to a greater increase in pleasure). Lines in red correspond to corticolimbic regions; lines in blue correspond to corticostriatal regions, and lines in green correspond to DMN regions. * indicates the right hippocampus.

**Correlations Between Change in ROI Activation with Clinical Change.**

TEPS-A and TEPS-C subscales showed a similar pattern of correlations as the TEPS Total score. MADRS post-pre change shows a similar pattern of correlations as the CDRS-R % change. See Supplementary Table 3 and Supplementary Figure 4.

Supplementary Figure 4.

***
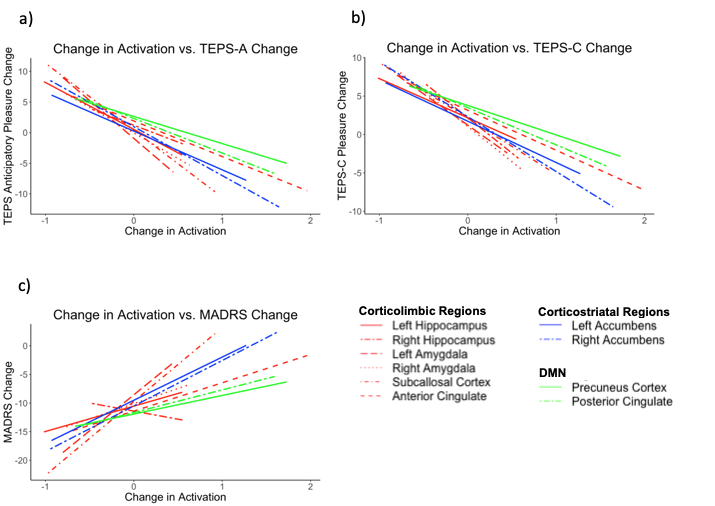
***

Note. Plots show the correlations between the average post-pre change in activation across conditions (congruent negative, congruent positive, incongruent negative, and incongruent positive) MADRS, and TEPS-A and TEPS-C. Lines in red correspond to corticolimbic regions; lines in blue correspond to corticostriatal regions, and lines in green correspond to DMN regions.

|  | | | | | | | | | |  |  |  |  |  |  |  |  |  |  |  |
| --- | --- | --- | --- | --- | --- | --- | --- | --- | --- | --- | --- | --- | --- | --- | --- | --- | --- | --- | --- | --- |
| Condition | Congruent Negative | | | | | Congruent Positive | | | | | Incongruent Negative | | | | | Incongruent Positive | | | | |
| Neural Network/Brain Region | CDRS % Change | MADRS  Change | TEPS-A  Change | TEPS-C  Change | TEPS Total Change | CDRS % Change | MADRS  Change | TEPS-A  Change | TEPS-C  Change | TEPS Total Change | CDRS % Change | MADRS  Change | TEPS-A  Change | TEPS-C  Change | TEPS Total Change | CDRS % Change | MADRS  Change | TEPS-A  Change | TEPS-C  Change | TEPS Total Change |
| **Corticostriatal Network** |  |  |  |  |  |  |  |  |  |  |  |  |  |  |  |  |  |  |  |  |
| Left Accumbens | -0.50 | 0.50 | -0.42 | -.72* | -0.54 | -0.40 | 0.42 | -0.26 | -0.39 | -0.32 | -0.53 | 0.49 | -0.38 | -.61* | -0.48 | -0.42 | 0.40 | -0.42 | -.71* | -0.54 |
| Right Accumbens | -0.34 | 0.33 | -0.48 | -.78** | -.60* | -0.41 | 0.42 | -0.33 | -0.56 | -0.42 | -.61* | .62* | -0.50 | -.81** | -.63* | -0.26 | 0.29 | -0.29 | -.66* | -0.43 |
| **Default Mode Network** |  |  |  |  |  |  |  |  |  |  |  |  |  |  |  |  |  |  |  |  |
| Posterior Cingulate | -0.04 | 0.05 | -0.19 | -0.49 | -0.30 | -0.05 | 0.15 | -0.22 | -0.30 | -0.25 | -0.32 | 0.34 | -0.45 | -0.58 | -0.51 | -0.20 | 0.29 | -0.28 | -0.47 | -0.36 |
| Precuneus | -0.04 | 0.04 | -0.18 | -0.44 | -0.28 | 0.01 | 0.08 | -0.14 | -0.15 | -0.15 | -0.26 | 0.29 | -0.39 | -0.52 | -0.45 | -0.26 | 0.31 | -0.25 | -0.43 | -0.32 |
| **Limbic** |  |  |  |  |  |  |  |  |  |  |  |  |  |  |  |  |  |  |  |  |
| Left Amygdala | -0.35 | 0.40 | -0.53 | -.66* | -0.59 | -0.10 | 0.20 | -0.05 | -0.05 | -0.06 | -0.60 | 0.57 | -0.47 | -0.59 | -0.53 | -0.27 | 0.17 | -0.25 | -0.38 | -0.30 |
| Left Hippocampus | -0.22 | 0.11 | -0.34 | -0.57 | -0.44 | -0.16 | 0.08 | -0.05 | 0.00 | -0.03 | -0.47 | 0.35 | -0.41 | -0.48 | -0.45 | -0.29 | 0.14 | -0.36 | -0.47 | -0.41 |
| Right Amygdala | -0.06 | 0.19 | -0.44 | -.64* | -0.53 | -0.06 | 0.23 | -0.21 | -0.47 | -0.31 | -0.09 | 0.14 | -0.28 | -.62* | -0.41 | 0.05 | 0.09 | 0.00 | -0.34 | -0.12 |
| Right Hippocampus | 0.07 | -0.01 | -0.31 | -0.55 | -0.40 | 0.42 | -0.39 | 0.23 | 0.03 | 0.16 | 0.03 | 0.06 | -0.27 | -0.57 | -0.39 | 0.12 | -0.05 | 0.05 | -0.26 | -0.06 |
| Subcallosal Cingulate | -0.39 | 0.55 | -0.56 | -.66* | -.62* | -0.34 | 0.46 | -0.30 | -0.29 | -0.31 | -0.53 | .60* | -0.41 | -0.59 | -0.49 | -0.39 | 0.48 | -0.47 | -.69* | -0.56 |
| Anterior Cingulate | -0.15 | 0.18 | -0.28 | -0.58 | -0.40 | -0.30 | 0.38 | -0.45 | -.618* | -0.53 | -0.46 | 0.44 | -0.41 | -.67* | -0.52 | -0.24 | 0.29 | -0.33 | -.63* | -0.45 |
| Numbers represent Pearson’s Correlations. * indicates p < .05 | | | | |  |  |  |  |  |  |  |  |  |  |  |  |  |  |  |  |

Supplementary Table 3. Change in Activation and Clinical Change

**Correlations Between Baseline ROI Activation with Clinical Change.**

Supplementary Table 4. Baseline Activation and Clinical Change

|  | | | | | | |  |  |  |  |  |  |  |  |  |  |  |  |  |  |
| --- | --- | --- | --- | --- | --- | --- | --- | --- | --- | --- | --- | --- | --- | --- | --- | --- | --- | --- | --- | --- |
| Condition | Congruent Negative | | |  |  | Congruent Positive | | |  |  | Incongruent Positive | | |  |  | Incongruent Negative | | |  |  |
| Neural Network/Brain Region | CDRS % Change | MADRS  Change | TEPS-A  Change | TEPS-C  Change | TEPS Total Change | CDRS % Change | MADRS  Change | TEPS-A  Change | TEPS-C  Change | TEPS Total Change | CDRS % Change | MADRS  Change | TEPS-A  Change | TEPS-C  Change | TEPS Total Change | CDRS % Change | MADRS  Change | TEPS-A  Change | TEPS-C  Change | TEPS Total Change |
| Corticostriatal Network |  |  |  |  |  |  |  |  |  |  |  |  |  |  |  |  |  |  |  |  |
| Left Accumbens | 0.36 | -0.29 | 0.21 | 0.53 | 0.33 | 0.32 | -0.24 | 0.26 | 0.33 | 0.30 | 0.37 | -0.36 | 0.35 | 0.55 | 0.44 | 0.37 | -0.33 | 0.35 | 0.44 | 0.39 |
| Right Accumbens | 0.35 | -0.30 | 0.36 | 0.54 | 0.44 | 0.24 | -0.15 | 0.13 | 0.17 | 0.15 | 0.41 | -0.41 | 0.39 | 0.60 | 0.48 | 0.29 | -0.28 | 0.20 | 0.34 | 0.26 |
| Default Mode Network |  |  |  |  |  |  |  |  |  |  |  |  |  |  |  |  |  |  |  |  |
| Posterior Cingulate | 0.24 | -0.15 | 0.31 | .62* | 0.43 | 0.23 | -0.25 | 0.46 | 0.37 | 0.44 | 0.20 | -0.25 | 0.46 | 0.55 | 0.51 | 0.16 | -0.18 | 0.27 | 0.40 | 0.33 |
| Precuneus | 0.22 | -0.14 | 0.28 | 0.55 | 0.38 | 0.22 | -0.28 | 0.37 | 0.20 | 0.32 | 0.14 | -0.23 | 0.40 | 0.46 | 0.44 | 0.16 | -0.19 | 0.16 | 0.28 | 0.21 |
| Corticolimbic |  |  |  |  |  |  |  |  |  |  |  |  |  |  |  |  |  |  |  |  |
| Left Amygdala | 0.46 | -0.40 | 0.52 | .64* | 0.58 | 0.44 | -0.50 | 0.38 | 0.34 | 0.38 | 0.47 | -0.41 | 0.39 | 0.49 | 0.44 | 0.38 | -0.24 | 0.26 | 0.32 | 0.29 |
| Left Hippocampus | 0.27 | -0.15 | 0.42 | .61* | 0.50 | 0.24 | -0.17 | 0.31 | 0.27 | 0.31 | 0.33 | -0.26 | 0.40 | 0.47 | 0.44 | 0.15 | -0.02 | 0.31 | 0.35 | 0.34 |
| Right Amygdala | 0.21 | -0.29 | 0.33 | 0.53 | 0.41 | 0.25 | -0.32 | 0.21 | 0.43 | 0.29 | -0.12 | 0.10 | -0.09 | 0.22 | 0.02 | 0.02 | -0.03 | -0.20 | 0.13 | -0.09 |
| Right Hippocampus | 0.06 | -0.08 | 0.27 | 0.50 | 0.36 | -0.08 | 0.13 | 0.05 | 0.30 | 0.14 | -0.28 | 0.22 | -0.16 | 0.11 | -0.07 | -0.16 | 0.18 | -0.29 | 0.06 | -0.18 |
| Subcallosal Cingulate | .65* | -.71* | .67* | .70* | .70* | .64* | -.62* | 0.56 | 0.41 | 0.53 | 0.53 | -0.57 | 0.50 | 0.60 | 0.55 | 0.50 | -0.58 | 0.57 | 0.57 | 0.59 |
| Anterior Cingulate | 0.50 | -0.37 | 0.33 | .64* | 0.45 | 0.56 | -0.45 | 0.51 | 0.55 | 0.54 | 0.55 | -0.47 | 0.50 | .74** | .61* | 0.53 | -0.40 | 0.36 | 0.51 | 0.43 |
| Numbers represent Pearson’s Correlations. * indicates p < .05 | | | | | |  |  |  |  |  |  |  |  |  |  |  |  |  |  |  |

See Supplementary Figure 5 and Supplementary Table 4. Greater baseline corticolimbic and corticostriatal activation across all four conditions showed medium to very large correlations with improved depression and anticipatory and consummatory pleasure. The corticolimbic regions showed the strongest correlations in the congruent negative condition. The corticostriatal network showed the most consistent pattern of correlations across brain regions in the incongruent negative condition. Greater baseline DMN activation in the congruent negative, congruent positive, and incongruent negative showed medium to large correlations with improved anticipatory and consummatory pleasure. Baseline activation to the incongruent positive condition showed small to medium correlations with anticipatory and consummatory pleasure in the same direction. Greater baseline DMN activation showed small correlations with improved depression. Similar to the relationships with change in brain activation, greater baseline corticolimbic and DMN activation was associated with greater clinical improvement.

Supplementary Figure 5.

***
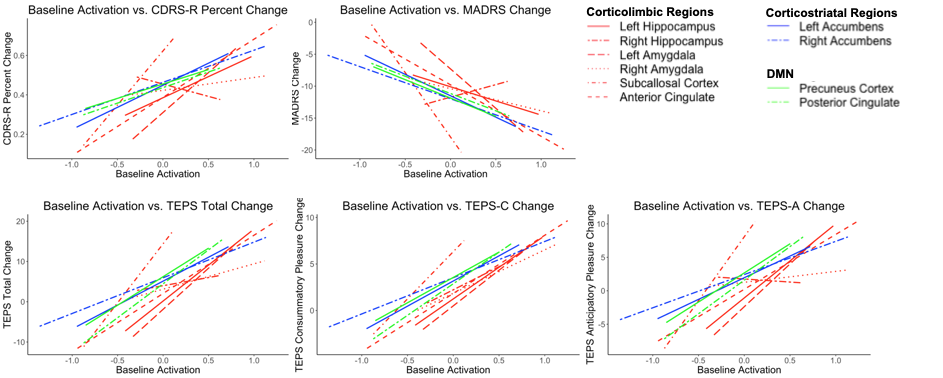
***

Note. Plots show the correlations between the average baseline activation across conditions (congruent negative, congruent positive, incongruent negative, and incongruent positive) and change in a) CDRS-R percent change, b) MADRS change, c) TEPS Total Change, d) TEPS-A change, and e) TEPS-C change. Lines in red correspond to corticolimbic regions; lines in blue correspond to corticostriatal regions, and lines in green correspond to DMN regions.
